# Production of Porous Films Based on Biodegradable Polyesters by the Casting Solution Technique Using a Co-Soluble Porogen (Camphor)

**DOI:** 10.3390/polym12091950

**Published:** 2020-08-28

**Authors:** Anatoly Nikolayevich Boyandin, Ljublyana Mikhailovna Dvoinina, Aleksey Grigorievich Sukovatyi, Anna Alekseevna Sukhanova

**Affiliations:** 1 Reshetnev Siberian State University of Science & Technology, 31 Krasnoyarsky Rabochy Av., 660037 Krasnoyarsk, Russia; shumilova.ann@mail.ru; 2 Institute of Biophysics of Siberian Branch of Russian Academy of Sciences, Federal Research Center “Krasnoyarsk Science Center SB RAS”, 50/50 Akademgorodok, 660036 Krasnoyarsk, Russia; a.sukovatiy@yandex.ru; 3 Siberian Federal University, 79 Svobodnyi Av., 660041 Krasnoyarsk, Russia; slav.lyana@yandex.ru

**Keywords:** polyhydroxybutyrate, polycaprolactone, biopolymers, polyesters, porosity, co-soluble porogen, camphor

## Abstract

Porous films have been prepared from degradable polymers—poly-3-hydroxybutyrate (PHB), poly-ε-caprolactone (PCL) and a blend of these polymers (1:3)—by adding porogen (camphor) to the polymer solution at 10%, 30% or 50% of the total mass of the polymer and porogen, and leaching it out afterwards. After the rinse, camphor content in films decreased to about 0.025%. The structure, physical/mechanical and biological properties of the films were investigated as dependent on their composition and porosity, which varied depending on the amount of camphor added. The surface of PHB films was porous, the PCL films were relatively smooth, and the PHB/PCL films had an intermediate structure. The addition of camphor increased the thickness (from 35 to 45 µm, from 40 to 80 µm and from 20 to 65 µm for PHB, PCL and PHB/PCL, respectively) and porosity (from 4.2(±3.6)% to 50.0(±12.8)%, from 6.4(±5.5)% to 54.5(±6.0)% and from 4.9(±4.8)% to 51.5(±5.8)%, respectively) of the films. The introduction (and removal) of 10% camphor into the PHB and PHB/PCL films led to an approximately twofold increase in the polar component of the free surface energy (from 5.4 ± 0.38 to 11.8 ± 1.33 and from 2.7 ± 0.13 to 5.2 ± 0.09 mN/m, respectively) but in other cases, on the contrary, a decrease in this indicator was registered. The increase of camphor addition from 0% to 50% gradually impaired mechanical properties of the films: so, Young’s modulus decreased from 3.6 to 1.8 GPa, from 0.30 to 0.12 GPa and from 0.50 to 0.20 GPa for PHB, PCL and PHB/PCL, respectively. At the same time, the water vapor transmission rate considerably increased from 197.37 ± 23.62 to 934.03 ± 114.34 g/m^2^/d for PHB films; from 1027.99 ± 154.10 to 7014.62 ± 280.81 g/m^2^/d for PCL films; and from 715.47 ± 50.08 to 4239.09 ± 275.54 g/m^2^/d for PHB/PCL films. Results of biocompatibility testing in the culture of NIH 3T3 mouse fibroblast cells showed that for the most of experimental samples cell adhesion and proliferation were comparable or superior to the corresponding parameters on the initial nonporous films. The best results were obtained for PHB films where at Day 3 of the experiment the registered cell density for experimental samples arrived at 2.66(±0.26) × 10^5^ cells/cm^2^ versus 1.29(±0.33) × 10^5^ cells/cm^2^ in the control. So, the proposed method can be used to construct highly porous cell scaffolds for cellular engineering.

## 1. Introduction

Biodegradable polymers are materials used in biomedical applications [1], for the production of degradable packaging [2], and in agriculture [3]. Polyhydroxyalkanoates (PHAs) hold a unique position among degradable biomaterials. PHAs are microbial polymers of hydroxy-derived fatty acids, which are thermoplastic, biocompatible and degradable in biological media [4]. Their properties make them highly perspective for a wide spectrum of biomedical applications including cell scaffolds for tissue engineering, implantable medical devices, patches for wound repair, systems for drug release, etc. [5]. PHAs are represented by polymers consisting of various monomer units and having diverse physicochemical properties: from highly crystalline thermoplastic materials to rubber-like elastomers [6,7]. The homopolymer poly-3-hydroxybutyrate (PHB) is the most extensively studied PHA. This thermoplastic polymer has high crystallinity (above 70%), and products fabricated from it are brittle and prone to aging, which limits their processing and usage. PHA copolymers, which consist of different monomer units, are more readily processable, but synthesis of these PHA types requires rather complicated approaches including special fermentation conditions and complex nutrient media or construction of genetically modified strains [8,9].

A promising approach to modifying and improving the properties of poly-3-hydroxybutyrate is to prepare PHB blends with different materials. One of such materials is poly-ε-caprolactone (PCL), a synthetic resorbable polymer, which is commonly used for prototyping [10] and is proposed as a material for biomedicine—to fabricate implants [11], cell scaffolds [12], systems for delivering drugs and bioactive substances [13], etc. The large size of PCL molecules and their linear chain structure enable manufacture of strong and elastic filaments, fibers and films, which can be used to produce pliable and mechanically strong constructs. PCL is also well known for its biocompatibility. However, being very hydrophobic, PCL does not favor cell adhesion and proliferation on the surface of cell scaffolds produced from PCL [14].

Cell scaffolds must have good adhesive properties and porous surface, which should favor cell attachment and proliferation. Another necessary condition is that the material and its degradation products must not cause adverse responses in cells [15]. The structure of the polymer construct is critical for cell adhesion and proliferation. For instance, rough surfaces can enhance integration, proliferation and differentiation of the cultured cells [16]. Interconnected porosity enables cell migration and proliferation and vascularization of cell scaffolds in the bulk tissue [17] and improves implant-tissue mechanical bonding [18].

Porous structures can be produced using various methods such as introducing of micronized crystalline agents into polymer systems [19,20], lyophilization of frozen solutions [21] and thermally induced phase separation [22]. However, no sufficiently strong porous structures with stable physical/mechanical and structural properties have been produced so far.

One approach to fabricating entirely porous constructs is to add a porogen to the polymer solution and then leach it out using a solvent that does not dissolve the polymer. The porogen must be nontoxic and soluble in polar and nonpolar solvents and enable formation of homogenous structures from the solutions. One of the substances meeting these requirements is camphor—a volatile crystalline substance that is readily soluble in various organic solvents, both organohalogen ones (used to produce polyester solutions) and alcohols (which do not dissolve polyesters and can, thus, be used to remove camphor from the mixes). In moderate dosage, camphor is not toxic and can be used for medical purposes [23].

The purpose of this study was to prepare porous constructs from degradable polymers using camphor as a porogen, to study their properties, and to test them as potential cell scaffolds.

## 2. Materials and Methods

### 2.1. Material

Poly-3-hydroxybutyrate (PHB; weight average molecular weight (M_w_) 720 kDa, polydispersity (Đ) 3.60, density (d) 1.25 g/cm^3^) was produced in the Siberian Federal University by microbial biosynthesis with the strain *Cupriavidus eutrophus* B-10646 using BioFlo 115 laboratory fermentor with a 14 L fermentation vessel (New Brunswick Scientific, Edison, NJ, USA) [24]. Poly-ε-caprolactone (PCL; M_w_ = 169 kDa, Đ = 1.86, d = 1.145 g/cm^3^) was manufactured by Sigma-Aldrich (Saint Louis, MO, USA). DL-Camphor (98% pure) was supplied by Career Henan Chemical Co. Ltd. (Zhengzhou, China). High-purity solvents chloroform and isopropyl alcohol were produced by EKOS-1 (Staraya Kupavna, Moscow Oblast, Russia).

### 2.2. Preparation of Films

PHB and PCL were separately dissolved in chloroform to prepare 2% (*w*/*v*) solutions. To produce the PHB:PCL 1:3 (*w*/*w*) blend the solutions were mixed in this ratio, and the mixture was kept for three hours with periodic stirring. Camphor was added to the polymer solutions at 10%, 30% or 50% of the total mass of the polymer and porogen contained in the solution. The mixtures were used to prepare films by the solvent evaporation technique, with 20 mL of the solution preheated to 35 °C and placed onto a degreased Petri dish. The films were dried at ambient temperature for 3 days in a laminar flow cabinet. After the solvent was completely evaporated, the mass of the polymer (or polymer blend) in each film was about 400 mg, with camphor content of 45 mg, 172 mg and 400 mg per film with initially added 10%, 30% or 50% camphor, respectively.

Porogen (camphor) was removed by sequentially rinsing it four times in isopropyl alcohol, and the mass loss was controlled. Residual camphor concentrations were measured by chromatography-mass spectrometry of the leaching solution (Agilent 7890A/5975C Inert, Agilent Technologies, Santa Clara, CA, USA), using reference camphor solutions to plot the calibration curve. Porous films with different camphor contents—10 (C10), 30 (C30) and 50 (C50) were investigated; non-porous polymer films (C0) were used as the reference films.

### 2.3. Methods of Investigating the Films

The surface microstructure of the films was studied with an S-5500 scanning electron microscope (Hitachi, Tokyo, Japan) after preliminary sputtering platinum by an Emitech K575X sputter coater (10 mA, 20 s—twice) (Quorum Technologies Ltd., Ashford, Kent, UK).

Surface properties of the films were tested with a DSA-25E drop shape analyzer (Krüss, Hamburg, Germany) using software DSA-4 for Windows. Drops of water and diiodomethane, 1.5 µL each, were alternately placed on the film surface, and contact angles (CA) of these liquids were measured in a semiautomatic mode, by the built-in “circle” method. The results of measurements were used to calculate surface free energy (SFE) and its dispersive (DSFE) and polar (PSFE) components by the Owens, Wendt, Rabel and Kaelble method [25,26].

Porosity was determined using the method proposed by Kavya et al. [27] and modified to analyze films. The dry films were weighed and immersed in ethanol for 5 min under vacuum. Then the films were taken out, excess ethanol was removed with filter paper, and the films were weighed again. Porosity (*P*) was determined using the following formulas:

$$P=\frac{V_{ethanol}}{V_{specimen}+V_{ethanol}};$$

$$V_{ethanol}=\frac{W_{w}-W_{d}}{d_{ethanol}};$$

$$V_{specimen}=\frac{W_{d}}{d_{polymer}}.$$

Here *W_d_* and *W_w_* are weights of the dry and moist samples, respectively, *d_ethanol_* is ethanol density—0.789 g/cm^3^, *d_polymer_* is polymer density—1.25 g/cm^3^ for PHB and 1.145 g/cm^3^ for PCL.

Physical-mechanical properties of the films were analyzed using an electromechanical tensile testing machine Instron 5565 (High Wycombe, UK). The thickness of films was measured prior to testing, by an electronic digital micrometer LEGIONER EDM-25-0.001 (China). Samples were maintained under normal conditions for at least two weeks to reach equilibrium crystallization. At least five samples were tested for each type of films. Measurements were conducted at room temperature; dumbbell-shaped samples 75 mm long and 13 mm wide were prepared, and the clamping length of the samples was 40 mm. The speed of the crosshead was 3 mm/min at room temperature. Young’s modulus (E, MPa), tensile strength (P, MPa) and elongation at break (ε, %) were automatically calculated by the Instron software (Bluehill 2, Elancourt, France). To obtain Young’s modulus, the software calculated the slope of each stress-strain curve in its elastic deformation region. Measurement error did not exceed 10%.

Water vapor transmission rate of the films (WVTR, gm/m^2^/d) was measured using a Mocon Permatran W system for measuring water vapor transmission rate (Minneapolis, MN, USA). Measurements were performed at a temperature of 37.8 °C and humidity of 100%. The area of each sample was 5 cm^2^. Each specimen was placed into a test cell, which was divided into two chambers separated by sample material. The inner chamber was filled with nitrogen (carrier gas) and the outer chamber with water vapor (test gas). Water molecules diffused through the sample material into the inner chamber and were conveyed to the sensor by the carrier gas. The test was stopped when water vapor concentration in the inner chamber was below the 3% deviation over a preset number of measurements.

To evaluate adhesive properties of the surface of the films and reveal potential cytotoxicity, disks of 15 mm diameter were cut out with a mold cutter and placed into 24-well plates (Techno Plastic Products AG, Trasadingen, Switzerland). The samples were preliminarily sterilized with 70% ethanol for 30 min and then washed once with incomplete Dulbecco’s Modified Eagle Medium (DMEM) (Gibco; Thermo Fisher Scientific, Inc., Waltham, MA, USA). NIH 3T3 mouse fibroblast cells were seeded onto the polymer disks (50,000 cells per well/mL). Cells were counted in the Goryaev chamber; a 0.4% trypan blue solution was added to cell suspensions to reveal dead cells. Polystyrene of the culture plate was used as the control.

Cells were cultivated in DMEM medium supplemented with 10% fetal bovine serum and a 1% Antibiotic-Antimycotic solution (Gibco; Thermo Fisher Scientific, Inc., Waltham, MA, USA) in a 5% CO_2_ atmosphere at a temperature of 37 °C for 7 days. The medium was replaced every three days. Cell viability was assessed in an 3-(4,5-dimethylthiazol-2-yl)-2,5-diphenyl-tetrazolium bromide (MTT) assay, which is based on the ability of dehydrogenases of live cells to reduce unstained forms of 3-(4,5-dimethylthiazol-2-yl)-2,5-diphenyl-tetrazolium bromide (MTT) (Sigma-Aldrich, Saint Louis, MO, USA) to crystals of blue formazan soluble in dimethyl sulfoxide (DMSO) (MP Biomedicals, Irvine, CA, USA). MTT assay was performed at Days 1, 3 and 7. In the plate with cells cultured on the films, the nutrient medium was replaced by the fresh one (950 µL) and 50 µL of MTT was added to it; the plate was incubated in a chamber thermostatically set to specific conditions at CO_2_ level maintained at 5% and at a temperature of 37 °C for 4 h. After incubation, the medium and MTT were replaced by DMSO (150 µL) to dissolve MTT-formazan crystals. Then the supernatant was transferred to the 96-well plate (TPP, Switzerland). Optical density was measured at a 490 nm wavelength with an iMark microplate absorbance reader (BioRad LABORATORIES Inc., Hercules, CA, USA). The number of viable, metabolically active cells was determined using the calibration curve.

### 2.4. Statistical Analysis

All experiments were carried out in at least five replicates. Statistical analysis of surface properties of the films was done by using built-in methods of DSA-4 software. Other data were handled using Microsoft Excel 2003 and expressed in the form of arithmetic means ± confidence intervals. Significant differences between mean values were studied using independent sample Student’s *t* test for independent samples (significance level: *p* = 0.1).

## 3. Results

**Figure 1:**
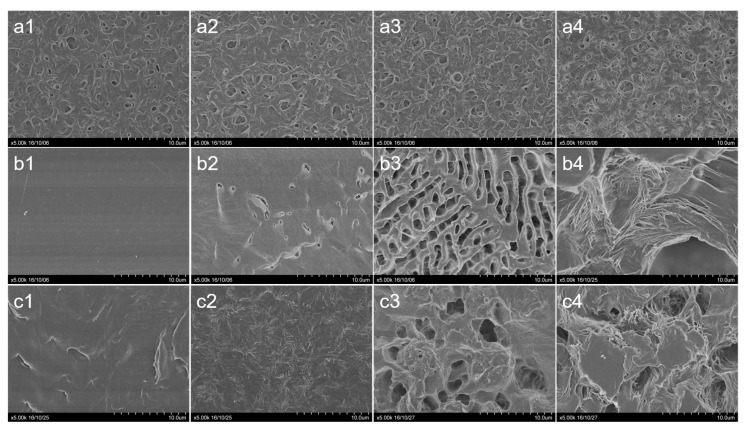
SEM images of the films (camphor contents before rinsing are given): (**a1**)—PHB (C0) (control); (**a2**)—PHB, camphor 10% (C10); (**a3**)—PHB, camphor 30% (C30); (**a4**)—PHB, camphor 50% (C50); (**b1**)—PCL, C0 (control); (**b2**)—PCL, C10; (**b3**)—PCL, C30; (**b4**)—PCL, C50; (**c1**)—PHB/PCL, C0 (control); (**c2**)—PHB/PCL, C10; (**c3**)—PHB/PCL, C30; (**c4**)—PHB/PCL, C50. PHB—poly-3-hydroxybutyrate; PCL—poly-ε-caprolactone.

**Figure 2:**
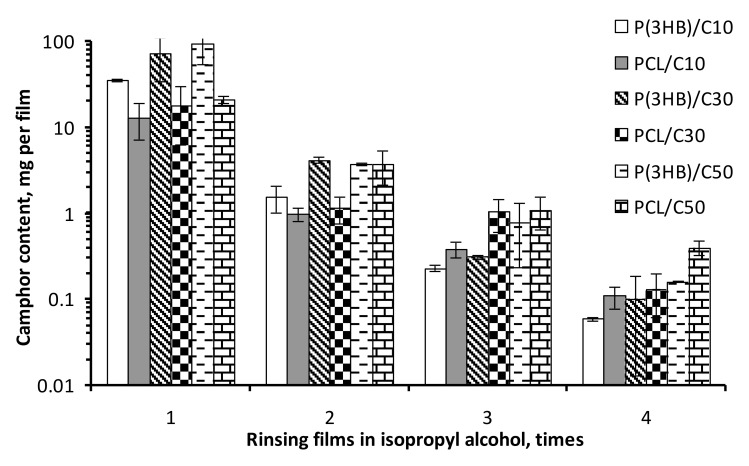
Camphor concentration decrease in the films rinsed in isopropyl alcohol four times.

The films of PHB, PCL and the PHB/PCL blend prepared without addition of camphor showed microscopic differences in their surface structure (Figure 1). The surface of PHB films was porous, the PCL films were smooth and the PHB/PCL films had small asperities on the relatively smooth surface. Preparation of porous films included addition of camphor to initial polymer solution and its subsequent removal by rinsing the films in isopropyl alcohol. Each rinsing event decreased camphor concentration in the films by approximately one order of magnitude (Figure 2). After the fourth rinse, camphor content reliably decreased to 0.1 mg/film, or about 0.025% of the film weight. That was far below camphor concentration toxic to humans (>20 mg/kg) [28].

As camphor concentration in the PHB films at the preparation stage was increased, considerably more pores and microfractures were formed and they were larger than those observed on the initial sample although the general surface topography remained the same (Figure 1, row 1). In the PCL films, which were smooth at microscopic level in the control (without camphor), leaching of camphor led to formation of a network of irregularly located pores (after adding 10–30% of the porogen the surface near pores remained smooth), whose density and size were increased with increasing porogen concentration (Figure 1, row 2). The similar pattern was observed for pores developed in the PHB/PCL films but they were more rounded and uniformly distributed (Figure 1, row 3).

The C10 films of PHB and PHB/PCL exhibited values of water contact angle decreased by 10 degrees or more and an increase in the polar component of surface free energy by a factor of more than two (Table 1), i.e., they had higher hydrophilicity. As camphor concentration was increased to 30%, the hydrophilicity of the films decreased to the initial level, and at 50%, it was significantly lower than in the control films. PCL films showed a more considerable decrease in hydrophilicity, and this was also determined by camphor concentration.

Investigation of the physical/mechanical properties of the films showed an increase in their porosity, which approximately corresponded to the percentage of the added camphor (Table 2). Statistically significant changes were registered for all C30 and C50 samples. The strength of the porous films was moderately decreased. Under addition of 10% camphor, Young’s modulus (E) of the films did not significantly change (for PHB and PHB/PCL films) or slightly decreased (by about 25% for PCL films). Similar dynamics was observed for tensile strength (P). An increase in porogen concentration to 30% and 50% of the total mass of the sample caused tensile strength to drop by a factor of 1.5—2.4 and 1.9—3 compared to the control, respectively. Variations in elongation at break (ε) were less consistent: addition of 10% camphor was accompanied by moderate decrease of ε, whereas higher additions (e.g., PCL/C50 and PHB/PCL/C50) could have led to an increase in this parameter. Polymer composition had a more marked effect on the strength of the films than their porosity.

The change in the bulk structure of PHB and PCL films caused by addition of camphor led to changes in their water vapor transmission rate compared to the nonporous films (Table 2). The WVTR values considerably increased from samples C0 to samples C50: from 197.37 ± 23.62 to 934.03 ± 114.34 g/m^2^/d for PHB films; from 1027.99 ± 154.10 to 7014.62 ± 280.81 g/m^2^/d for PCL films; and from 715.47 ± 50.08 to 4239.09 ± 275.54 g/m^2^/d for PHB/PCL films.

**Figure 3:**
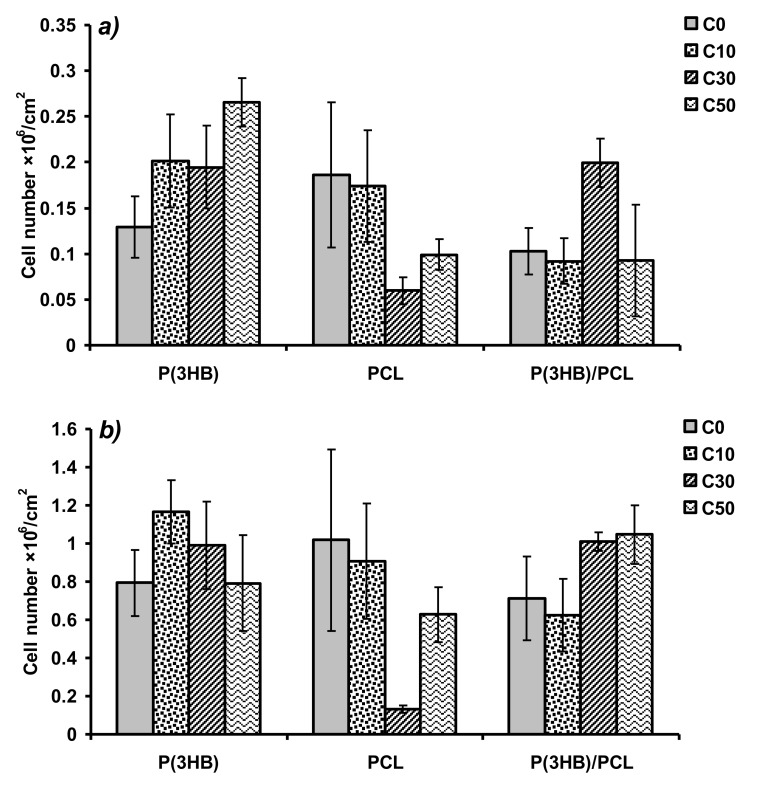
Results of MTT assay—the number of viable NIH 3T3 mouse fibroblast cells on polymer films at Days 3 (**a**) and 7 (**b**) of cultivation.

MTT assay showed higher fibroblast proliferation on porous PHB films than on control ones. At Day 3 of the experiment (Figure 3a), the best effect was registered for samples C50 (2.66(±0.26) × 10^5^ cells/cm^2^ versus 1.29(±0.33) × 10^5^ cells/cm^2^ in the control), but at Day 7 (Figure 3b), it was statistically significant for samples C10 only (1.17(±0.17) × 10^6^ cells/cm^2^ versus 0.79(±0.17) × 10^6^ cells/cm^2^ in the control). For samples PCL/C10, the level of fibroblast adhesion was comparable to the control level. For samples PCL/C30 cell adhesion was significantly decreased; for PCL/C50 the registered decrease was statistically insignificant. For PHB/PCL films, at Day 3, the highest cell proliferation rate was observed on samples C30 (2.00(±0.27) × 10^5^ cells/cm^2^ vs. 0.92–1.03 × 10^5^ cells/cm^2^ on the other samples). For samples C50, favorable effects was noted by Day 7 but they were not statistically significant.

## 4. Discussion

It is important for cell scaffolds and wound dressings to have interconnected porosity, which enables vascularization, sufficient flows of nutrients, gas and water exchange and waste outflow. At the same time, they should remain mechanically strong. A common method for preparing porous carriers is to supplement the solution with the pore-forming particles (sodium chloride, sucrose, etc.) that are insoluble in it and then leach them out. However, by using this method, it is difficult to prevent pore-forming particles from agglomerating, enable their uniform distribution and control mechanical properties of the product [19]. Another possible method is freeze-drying: freezing of the polymer solution followed by sublimation of the solvent from the frozen state by decreasing the pressure [21]. High porosity constructs can be produced by freezing polymer solution at temperatures between −20 and −196 °C followed by leaching the solvent out [29]. The low temperatures and the drying conditions needed to produce porous constructs using this method make it very power-consuming, which is a serious limitation to its use. This method directly depends on the testing and selection of several experimental parameters such as solution concentration, solvent type, freezing temperature, freezing rate and partial pressure [30]. A widely used technique now is thermally induced phase separation: rapid changes of the temperature of the polymer solution to a certain critical value, which results in spontaneous separation of the solution into phases and formation of pores in the bulk of the construct after solvent evaporation. By varying polymer concentrations and temperature, one can control micro- and macrostructure of the construct [22] and produce highly porous polymer membranes [31]. Limitations of this method are control issues and unstable pore sizes and porosity structure [32].

Polymers based on PHAs and PCL, which are highly biocompatible and slowly degraded, are promising materials for reconstructive bioengineering, construction of bio-artificial organs, and tissue and cellular engineering. An additional way to improve mechanical properties and overcome the drawbacks of pure polymers, to enhance their elasticity and mechanical strength is to prepare polymer-based blends [33,34]. As shown in a previous work [35], PCL with added 25% of poly-3-hydroxybutyrate-co-(7.5 mol. %)-3-hydroxyvalerate copolymer demonstrated better mechanical properties (e.g., elongation at break) than even pure PCL.

The porogen used in this study was a nontoxic, co-soluble camphor added to the polymer solution and then leached out from the polymer-porogen system.

We prepared different biopolymer films based on biodegradable polyesters (PHB and PCL), whose structures and water vapor transmission rates were changed by introducing porosity. Electron microscopy showed the greatest effects for PCL and PHB/PCL films although for PCL the morphology was insufficiently uniform.

The porosity values generally correlated with the amounts of the camphor initially added to the solution for preparing films. The polymer composition of the films had a weaker effect on their porosity, which varied from the values close to zero in the control samples to about 50% in the samples prepared with 50% porogen added to the polymer solution (Table 2). The structure of the films and their WVTR were determined by both polymer composition and porogen concentration. The WVTR increase correlated with microscopy data: for PHB films WVTR increased by a factor of 4.7, for PCL films by a factor of 6.8 and for PHB/PCL films by a factor of 5.9.

PHB and PHB/PCL porous biopolymer films did not produce any cytotoxic effects on the cultured fibroblast cells and favored cell proliferation. Among the PHB films, at Day 3, the most pronounced beneficial effect was observed on samples C50, which could be attributed to their high WVTR. This advantage, however, became less significant at Day 7, when samples C10 and, to a lesser extent, C30 showed better results. These delayed effects could be primarily associated with the surface hydrophilicity, which was highest for C10 (based on PSFE values, Table 1) and correlated well with results of the MTT assay.

For the PHB/PCL films, the more rapid growth of cells on C30 and, to a lesser extent, C50 films, could not be attributed to hydrophilic properties of the films. SEM images (Figure 1), however, show an obvious change in the topography of these films compared to the control and C10. In addition to hydrophilicity and pore structure, pore size is an important factor [36] for many cell effects. Formation of 1.5–4 µm uniformly distributed pores was only observed on the PHB/PCL/C30 and PHB/PCL/C50 films, which could be a factor stimulating cell growth. The porosity achieved in this study was much lower than in the studies that employed other methods of the treatment of polyesters. In a study by Conde et al. [37], NaCl crystals were added to the solution of poly-l-lactic acid (PLLA) causing formation of the structures with pores larger than 150 µm. The addition of gelatin particles to the PLLA solution in 1,4-dioxane enabled formation of 280–450 µm pores [38]. The use of the technique of phase separation in the PLA solution in ethanol/dichloromethane based on the preferential evaporation of the lower-boiling dichloromethane enabled production of polymer structures with pores larger than 100 µm. Matrices with smaller, 30–90 µm, pores were prepared by CO_2_ blowing followed by ultrasonic treatment [39]. Comparable results (30–100 µm) were obtained by using a similar technique (with no ultrasonic treatment) on poly(dl-lactide-co-glycolide) 85/15 [40]. Finally, a combination of solid state extrusion and porogen (NaCl) leaching method produced pores of diameter of about 9 µm, resulting from destruction of larger NaCl particles during the processing [41]. In general, the method used allows for a smaller pore size than after using insoluble porogens. However, the observed correlation between threshold content and pore size is complex and highly dependent on the composition of the polymer matrix (Figure 1). Thus, in the case of PHB matrices, the inclusion and subsequent leaching of camphor did not affect the pore size, although it significantly increased the total porosity and moisture absorption. In the case of PCL, an increase in camphor inclusions significantly increased the pore size. Mixed samples showed intermediate values.

In the case of PCL films, the C30 and C50 PCL samples showed the highest hydrophobicity (PSFE 0.1 and 1.9 mN/m), which was consistent with its lowest values in the MTT assay (Figure 3); the tendency observed at Day 3 had been exhibited until the end of the experiment. The other reason of such MTT results can be in nonuniform pore arrangement of the PCL porous films (especially for PCL/C30; Figure 1, row 2) which is normally a disadvantage for tissue healing in vivo as cells are more prone to colonize oriented structures due to the superior homogeneity in nutrients as oxygen distribution [42]. This problem can be possibly overridden to change or tune parameters of film preparation including type of solvent, temperature which can also affect evaporation time and polymer crystallization from the solution) and even porogen composition.

None of the experimental films except PCL/C30 and PCL/C50 had biological properties inferior to those of the control films, as suggested by results of the MTT assay. The main aim of adding porogen is to enhance air and water vapor permeability of films, which is an important factor in, e.g., constructing wound-healing dressings. At the same time, the biocompatibility of the material must be preserved at a level characteristic of the initial polymer; enhancing biocompatibility is a secondary, though desirable, goal. This condition was fully achieved at least for the PHB and PHB/PCL matrices.

So, the proposed method can be used to construct highly porous cell scaffolds for cellular engineering. In the future, this method can be employed to use co-soluble components (such as camphor) to develop porosity in combination with insoluble substances (such as NaCl or sucrose) to achieve macroporosity. Such two-level porosity could, on the one hand, facilitate more effective diffusion of nutrients and gases in the matrix layer and, on the other, produce a necessary spatial structure for cell growth in the bulk.

## Figures and Tables

**Table 1:** Measurements of contact angles and surface energy parameters of polymer films prepared with camphor added at different concentrations.

Polymer	Sample	CA_W_ (°)	CA_DIM_ (°)	SFE (mN/m)	PSFE (mN/m)	DSFE (mN/m)
PHB	C0	78.5 ± 7.33	48.5 ± 1.86	40.5 ± 0.76	5.4 ± 0.38	35.1 ± 0.38
PHB	C10	61.6 ± 17.32	38.3 ± 4.73	52.3 ± 2.38	11.8 ± 1.33	40.5 ± 1.05
PHB	C30	74.6 ± 6.28	41.2 ± 1.4	45 ± 0.65	6 ± 0.34	39 ± 0.31
PHB	C50	81.7 ± 5.31	43.2 ± 1.23	41.6 ± 0.49	3.6 ± 0.22	38 ± 0.26
PCL	C0	54.7 ± 7.12	20.4 ± 3.42	60.7 ± 1.44	13 ± 0.61	47.7 ± 0.82
PCL	C10	65.8 ± 5.56	23.6 ± 3.33	54.6 ± 1.19	7.9 ± 0.39	46.6 ± 0.79
PCL	C30	95.3 ± 6.12	24.6 ± 5.79	46.4 ± 1.43	0.1 ± 0.06	46.3 ± 1.38
PCL	C50	83.4 ± 2.62	26.5 ± 1.59	47.5 ± 0.47	1.9 ± 0.09	45.6 ± 0.37
PHB/PCL	C0	85.4 ± 2.71	44.8 ± 2.37	39.8 ± 0.63	2.7 ± 0.13	37.1 ± 0.5
PHB/PCL	C10	75.8 ± 1.56	38.1 ± 0.93	45.8 ± 0.3	5.2 ± 0.09	40.6 ± 0.21
PHB/PCL	C30	83.4 ± 1.27	35.6 ± 0.94	44.2 ± 0.27	2.5 ± 0.05	41.7 ± 0.21
PHB/PCL	C50	98.9 ± 4.2	35.1 ± 1.57	42 ± 0.38	0 ± 0.02	42 ± 0.36

CA_W_ and CA_DIM_—contact angles of water and diiodmethane, respectively; SFE—surface free energy; PSFE and DSFE—polar and disperse parts of SFE, respectively.

**Table 2:** Physical/mechanical properties and water vapor transmission rate of polymer films.

Polymer	Sample	Thickness (µm)	Porosity (%)	E (MPa)	P (MPa)	ε (%)	WVTR (g/m^2^/d)
PHB	C0	35	4.2 ± 3.6	3634.41 ± 284.20	40.11 ± 3.02	1.55 ± 0.09	197.37 ± 23.62
PHB	C10	35	11.3 ± 3.1	3516.88 ± 217.34	32.79 ± 1.03	1.08 ± 0.05	226.62 ± 27.19
PHB	C30	35	30.1 ± 10.3	2722.82 ± 88.23	24.18 ± 1.84	1.16 ± 0.30	232.00 ± 27.12
PHB	C50	45	50.0 ± 12.8	1815.64 ± 17.84	13.54 ± 0.60	0.9 ± 0.03	934.03 ± 114.34
PCL	C0	40	6.4 ± 5.5	302.15 ± 11.60	13.54 ± 0.42	55.37 ± 19.25	1027.99 ± 154.10
PCL	C10	60	11.0 ± 3.4	219.20 ± 4.28	10.70 ± 0.02	20.14 ± 4.65	1410.94 ± 134.36
PCL	C30	75	30.7 ± 6.4	114.24 ± 6.67	5.58 ± 0.15	26.93 ± 3.21	4674.51 ± 118.42
PCL	C50	80	54.5 ± 6.0	121.99 ± 6.29	5.62 ± 0.20	68.4 ± 2.53	7014.62 ± 280.81
PHB/PCL	C0	20	4.9 ± 4.8	502.07 ± 62.42	14.12 ± 1.55	45.69 ± 18.16	715.47 ± 50.08
PHB/PCL	C10	40	14.3 ± 4.8	562.32 ± 46.23	13.81 ± 1.29	33.94 ± 12.35	712.92 ± 47.10
PHB/PCL	C30	50	33.1 ± 4.4	359.57 ± 46.73	8.97 ± 1.36	48.00 ± 15.72	1608.00 ± 126.20
PHB/PCL	C50	65	51.5 ± 5.8	199.38 ± 15.59	7.37 ± 0.35	103.80 ± 12.99	4239.09 ± 275.54

E—Young’s modulus; P—tensile strength; ε—elongation at break; WVTR—water vapor transmission rate.
